# Characterizing Newly Repopulated Microglia in the Adult Mouse: Impacts on Animal Behavior, Cell Morphology, and Neuroinflammation

**DOI:** 10.1371/journal.pone.0122912

**Published:** 2015-04-07

**Authors:** Monica R. P. Elmore, Rafael J. Lee, Brian L. West, Kim N. Green

**Affiliations:** 1 Department of Neurobiology and Behavior, Institute for Memory Impairments and Neurological Disorders (UCI MIND), University of California, Irvine, Irvine, California, United States of America; 2 Plexxikon Inc., Berkeley, California, United States of America; University of Cologne, GERMANY

## Abstract

Microglia are the primary immune cell in the brain and are postulated to play important roles outside of immunity. Administration of the dual colony-stimulating factor 1 receptor (CSF1R)/c-Kit kinase inhibitor, PLX3397, to adult mice results in the elimination of ~99% of microglia, which remain eliminated for as long as treatment continues. Upon removal of the inhibitor, microglia rapidly repopulate the entire adult brain, stemming from a central nervous system (CNS) resident progenitor cell. Using this method of microglial elimination and repopulation, the role of microglia in both healthy and diseased states can be explored. Here, we examine the responsiveness of newly repopulated microglia to an inflammatory stimulus, as well as determine the impact of these cells on behavior, cognition, and neuroinflammation. Two month-old wild-type mice were placed on either control or PLX3397 diet for 21 d to eliminate microglia. PLX3397 diet was then removed in a subset of animals to allow microglia to repopulate and behavioral testing conducted beginning at 14 d repopulation. Finally, inflammatory profiling of the microglia-repopulated brain in response to lipopolysaccharide (LPS; 0.25 mg/kg) or phosphate buffered saline (PBS) was determined 21 d after inhibitor removal using quantitative real time polymerase chain reaction (RT-PCR), as well as detailed analyses of microglial morphologies. We find mice with repopulated microglia to perform similarly to controls by measures of behavior, cognition, and motor function. Compared to control/resident microglia, repopulated microglia had larger cell bodies and less complex branching in their processes, which resolved over time after inhibitor removal. Inflammatory profiling revealed that the mRNA gene expression of repopulated microglia was similar to normal resident microglia and that these new cells appear functional and responsive to LPS. Overall, these data demonstrate that newly repopulated microglia function similarly to the original resident microglia without any apparent adverse effects in healthy adult mice.

## Introduction

Microglia are the primary immune cell of the brain, detecting and responding to pathogens within the CNS [1–4]. In addition to their immunoprotective functions, microglia may also play critical roles in modulating neuronal numbers, structure, and connectivity during development [5–8], leading to the idea that they may also play similar roles in the adult and aged brain [5,9–12]. Microglia take up residence in the CNS during development and form a self-replenishing cell population with no contributions from peripheral cells [3,13–15]. Crucially, microglial dysfunction has been implicated in traumatic brain injury (TBI; [16,17]; aging (e.g., microglial senescence; [1,5,18–20]), and neurodegeneration ([21]; e.g., Alzheimer’s disease; [20,22,23]), and thus understanding the biology of these cells, along with ways to manipulate their numbers and biology, is vital to future treatment options [24].

The CSF1R is expressed by myeloid lineage cells, including monocytes and macrophages in the periphery [25,26], and microglia within the CNS [26,27], and is essential for microglia development and survival. For example, CSF1R knockout mice are born without microglia and show developmental deficits, including disrupted brain growth and olfactory deficits [14,26,27]. Interestingly, mutations in the CSF1R in humans has been linked to rare neurodegenerative disorders, such as hereditary diffuse leukoencephalopathy with spheroids (HDLS) [28,29]. We recently reported that inhibition of CSF1R in adult mice leads to the elimination of virtually all microglia within days, using the dual CSF1R/c-Kit inhibitor PLX3397 [30]. Given the lack of microglia in CSF1R knockout mice, the effects of PLX3397 on microglial elimination are likely due entirely to CSF1R inhibition, rather than c-Kit. Notably, microglia appear to be uniquely dependent on CSF1R signaling for their survival, as myeloid cells in the periphery are not substantially depleted using the same inhibitors [31–37]. We found that microglia remain eliminated from treated mice for as long as the inhibitor is given, albeit weeks or months, providing a novel tool for studying microglial function in the adult. Indeed, adult mice devoid of microglia for up to 2 months showed no behavioral or cognitive impairments [30]. Surprisingly, following the removal of the inhibitor, non-microglial cells proliferated and then switched on expression of microglia-associated genes, such as IBA1, CX3CR1, Tmem119, Siglech, Pu.1, and TREM2 [30], and then began to assume a microglial morphology, thus revealing a microglial progenitor within the adult CNS. The brain became fully repopulated with the same number of microglia as controls within 7–14 days [30].

Since these newly repopulated microglia have not been fully characterized, it is unknown what effects these cells have on behavioral and cognitive function, as well as their ability to respond to an inflammatory challenge. Systemic challenge with a bacterial mimetic, such as LPS, has been used extensively in the literature to investigate the impacts of peripheral infection on neuroinflammation and brain cell function [38–41]. These functions are important to characterize, as microglial activation and microglial-derived factors can modulate and impair cognition and long-term potentiation (LTP; [12,42,43]). Therefore, our goal in this study was to characterize the newly repopulated microglia in the adult mouse following CSF1R/c-Kit inhibitor removal and investigate the impacts of microglial repopulation on animal behavior, cell morphology, and neuroinflammation. We find that the repopulated microglia cause no changes in behavior, cognition, or motor function. However, these cells differ in many cell morphology markers, which appear to resolve over time, and are still responsive to LPS stimulation.

## Materials and Methods

### Ethics Statement

All experiments were carried out in strict accordance with the Guide for the Care and Use of Laboratory Animals of the National Institutes of Health and an approved animal research protocol (2011–3014) by the Institutional Animal Care and Use Committee (IACUC) at the University of California, Irvine (UCI). All efforts were made, especially during LPS challenge and euthanasia, to minimize animal suffering.

### Elimination of Microglia Using a CSF1R/c-Kit Inhibitor, Followed by Microglial Repopulation

A total of 24 two month-old wild-type (C57BL/6 background, raised in-house) mixed sex mice were used for this experiment (experimental unit = single mouse). Mice were randomly placed on either control diet (n = 10) or a diet to eliminate microglia (n = 14) for 21 days, using the inhibitor PLX3397 (290 mg/kg chow, as previously described [30]). These sample sizes have been shown to be sufficient to detect treatment differences in previous experiments in our lab. Mice were socially housed in standard sterile ventilation-top cages with corncob bedding and access to nesting material (i.e., compressed cotton squares and thin cardboard strips). Mice were maintained on a 12-hr light/dark cycle and the room was kept at 21.1°C (range 20–23.3°C). Mice were checked daily by animal care staff to ensure overall health and well-being. Following microglial elimination, four of the inhibitor-treated mice were sacrificed. Specifically, mice were anesthetized with Euthasol (Virbac) via intraperitoneal (IP) injection and unresponsiveness to physical stimulation was ensured prior to perfusion with PBS to prevent animal suffering. Following perfusion, brains were post-fixed in 4% paraformaldehyde for histology to confirm microglial elimination. The remaining microglia-eliminated mice were then placed on control diet for an additional 21 days to allow for microglial repopulation to occur (n = 10). Following behavioral testing (described below), control and repopulated mice were euthanized and tissue collected as described above.

### Cognitive Behavioral Testing

Control mice were compared to mice with 21 d repopulated microglia in tasks of behavior, cognition, and motor function, including: open field (test of motor function and anxiety), novel object (test of cortical learning and memory), rotarod (test of motor function), and Morris water maze with LPS administration prior to the start of reversal testing (test of hippocampal learning and memory). Testing was conducted on days 14–21 after inhibitor removal, when microglia had repopulated. *Handling (days 11–13)*: Mice were handled for 3 days prior to the start of behavioral testing to habituate the animals to the experimenter. *Open field (day 14)*: In brief, mice were placed in an opaque white box (33.7 cm L x 27.3 cm W x 21.6 cm H) for 5 min while their behavior was video recorded. The amount of time spent in the center versus the perimeter of the arena, as well as a motor readout (distance moved), was obtained. Increased thigmotaxis (“wall hugging”) behavior is indicative of increased anxiety in rodents [44–46]. *Novel object (days 15 and 16)*: Using the opaque boxes from the open field task, mice were allowed to freely explore 2 identical objects (either small glass beakers or plastic building blocks; counterbalanced for treatment) and their behavior was recorded for 5 min. The next day (24 h later), one of the familiar objects was replaced with a novel object (either beaker or block) and behavior was recorded for 3 min. The amount of time spent investigating the novel object was determined by calculating the discrimination index (time investigating new object—time investigating familiar object / total time) and is presented as a percentage, where chance level of investigation for each object is 50% of the testing time. Animals that better remember the familiar object from 24 h previously should show a higher preference, and thus, a higher percentage of time than chance interacting with the novel object. *Rotarod (day 16)*: The motor capabilities of the mice were tested using an accelerating rotarod (Ugo Basile). Each mouse was placed on the rotarod beam for a maximum of 5 min while it accelerated from 8 to 40 rpm. The experimenter stopped the timer when either the mouse fell off the beam or the mouse held on to the beam and its body completed two full rotations. A total of 5 trials were performed per mouse, each with a 15-min intertrial interval. The longer the mice stayed on the rod, the higher their motor abilities are said to be. *Morris water maze (days 17–21)*: For this task, a white opaque plastic circular pool (122 cm diameter, 35.5 cm tall) was filled with approximately 25 cm of tap water, which was heated to 21°C and made opaque with white paint (non-latex, nontoxic, and water soluble tempura paint). A white plastic platform (11.4 cm diameter, ~24 cm tall) with a grid overlay (to assist the mice in climbing/gripping) was submerged 0.5 cm below the surface of the water. Distinct two-dimensional visual cues were positioned around the perimeter of the pool. The pool was visually divided into four quadrants, and the hidden platform was placed in one of these quadrants (#2), where it remained throughout acquisition (days 17–19). During acquisition of this task, animals learn to use distal visuospatial cues to locate the hidden platform more efficiently over successive trials and testing days [47–49]. During acquisition, mice were divided into groups of four for individual testing. At the start of each testing session, mice were placed on the platform for 10 s. Using a pseudorandom pattern, whereby no quadrant was used more than once per testing session, mice were placed in the pool and allowed to swim freely for 60s or until the platform was located. If the platform was not located in this time, the mice were guided to the platform by the experimenter. In both cases, mice were allowed to remain on the platform for 10 s following completion of the trial. Each mouse in the group completed the first trial and rested in a holding cage under a heat lamp before continuing with the next trial. After all mice completed all four trials and had dried thoroughly under the heat lamps, they were returned to their home cages, and the next group of mice was tested. On day 20 (24 h after the last day of acquisition testing), the platform was removed and the mice were subjected to a 60 s probe trial to assess spatial memory for the platform location. The following day (day 21), half of the control mice and half of the repopulated microglia mice were injected IP with LPS, 0.25 mg/kg (n = 5) or PBS (n = 5), and reversal testing was performed 4 h later. During reversal testing, the hidden platform was moved to the opposite quadrant of the pool (#4), but all visual cues remained intact. Mice were placed on the platform for 30 s preceding the start of the first trial and given 4 trials to locate the platform in its new location. Reversal testing measures how quickly an animal is able to extinguish its knowledge of the previously learned platform location and acquire a direct path to the new location [47,48,50]. Unless otherwise stated, behavioral readouts for all tasks were calculated from video using the EthoVision XT tracking system (Noldus Information Technology). Mice were euthanized and tissue was collected at 6 h post LPS or PBS injection.

### Quantitation and Morphological Analysis of Microglia

Fluorescent immunolabeling of the microglia followed a standard indirect technique (primary antibody followed by fluorescent secondary antibody) as described in Neely et al. [51]. Brain tissue (sliced at 40 μm) was stained using the anti-ionized calcium-binding adapter molecule 1 (IBA1, polyclonal, rabbit) antibody (1:1000; Wako, Cat. #019–19741), mounted on slides, and coverslipped using Dapi Fluoromount-G (SouthernBiotech). Half brain images were obtained by stitching using a Zeiss AxioImager M2 upright microscope and Stereo Investigator software package from MicroBrightField. In addition, tissue was stained with anti-hexaribonucleotide binding protein-3 (NeuN, monoclonal, mouse) antibody (1:1000; Millipore; Cat. #MAB377) to label neurons and anti-glial fibrillary acidic protein (GFAP, polyclonal, chicken) antibody (1:500; Abcam; Cat. #ab4674) to label astrocytes, and 10x and 63x z-stack images obtained for each treatment using confocal microscopy.

For morphological analysis brain tissue was stained with both anti-IBA1 (as described above) and anti-NeuN (as described above) antibodies. Microglial cell counts were obtained by scanning regions at a 10x objective using comparable sections in each animal via confocal microscopy. Microglial morphology was assessed using these confocal images of the IBA1^+^ microglia within the cortical and hippocampal regions of the control vs. 21 d repopulated tissue. An additional group of mice were assessed (n = 5) at 14 d repopulation (animals were treated and brain tissue was collected as described above) to investigate the changes in microglial morphology over time following inhibitor removal. Microglia were modeled using Bitplane Imaris software and changes in microglial markers, such as cell body area, process diameter, process length, and number of microglial branches, were analyzed.

### Inflammatory Profiling

For inflammatory profiling of the microglia repopulated brain, half of the mice from each dietary treatment (control or 21 d PLX3397) were administered either LPS (0.25 mg/kg) or an equivalent volume of PBS via IP injection (n = 5 per group). A low dose of LPS, which would still initiate an inflammatory response, was used in this study to minimize animal suffering. Mice were euthanized 6 h post-injection (as described above and following reversal testing in the Morris water maze) and half of each brain was collected and snap frozen. RNA was extracted (RNA Plus Universal Mini Kit, Qiagen), cDNA synthesized (iScript kit, BioRad), and quantitative RT-PCR performed using a commercially available immune panel that tests 96 genes (including pro-inflammatory cytokines, chemokines, and microglial specific markers; Taqman Array Mouse Immune Panel; Invitrogen). mRNA expression data were analyzed using the comparative threshold cycle (Ct) method [52] and results are expressed as percent change from controls administered PBS.

### Statistical Analysis

Data were analyzed using unpaired student’s t-tests (control vs. repopulated) in Microsoft Excel or as a two-way ANOVA (control vs. repopulated and LPS vs. PBS, with “mouse” as the experimental unit) using the MIXED procedure of the Statistical Analysis Systems software (SAS Institute Inc.). The two-way ANOVA data were checked for adherence to statistical assumptions (i.e., homogeneity of variance, normality of residuals, etc. [53]), by plotting the residuals within SAS, and did not require transformation. Post-hoc paired contrasts were used to examine biologically relevant interactions from the two-way ANOVA regardless of statistical significance of the interaction. Data are presented as raw means ± standard error of the mean (SEM). For all analyses, statistical significance was accepted at P<0.05; statistical trends at P<0.10.

## Results and Discussion

### Elimination of Microglia Using a CSF1R/c-Kit Inhibitor, Followed by Microglial Repopulation

**Fig 1:**
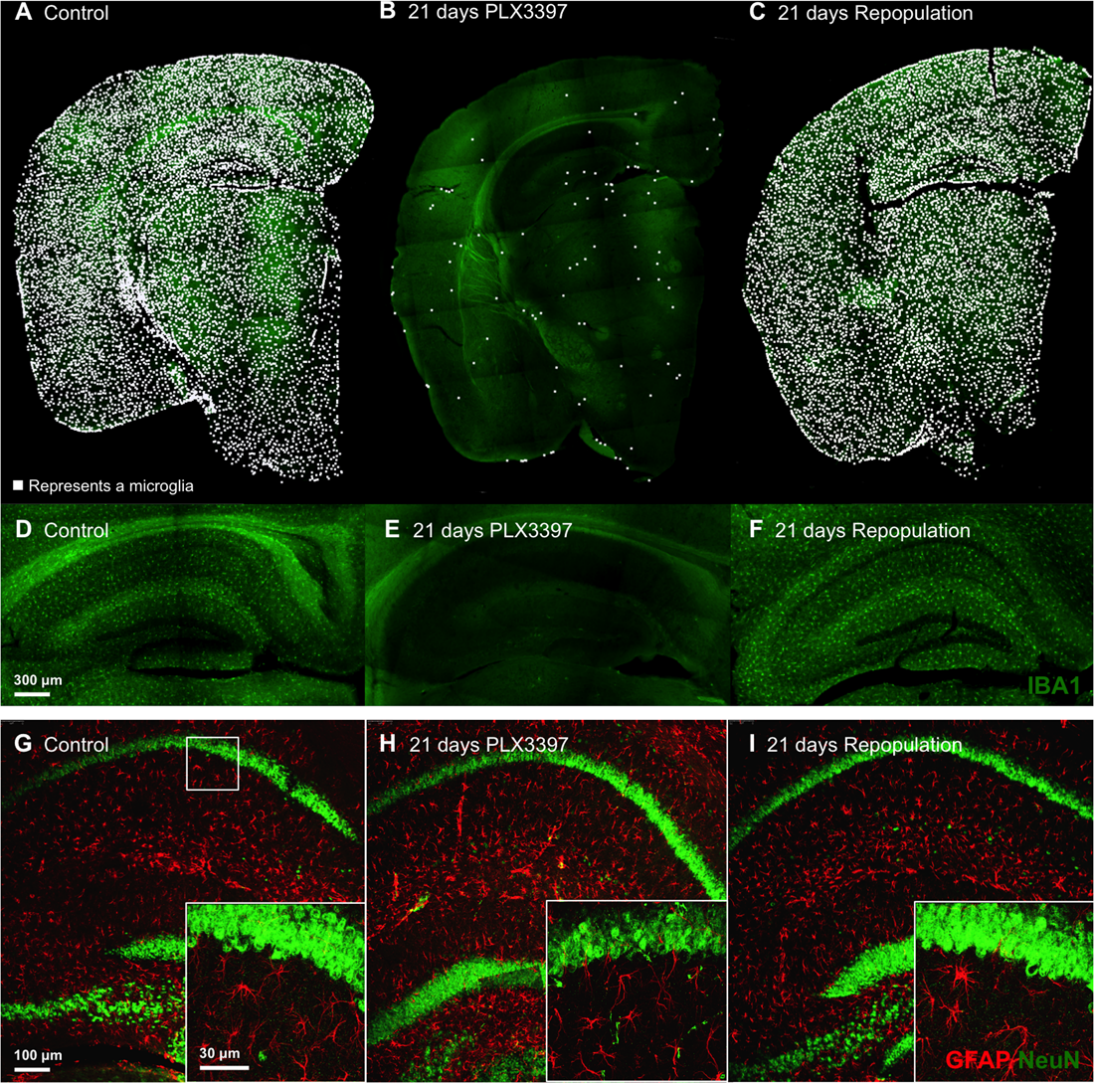
Elimination of Microglia in the Adult CNS Using CSF1R/c-Kit Inhibition, Followed by Repopulation with New Cells. A, B) Two month-old wild-type mice were placed on either control (n = 10) or inhibitor diet (PLX3397, provided at 290 mg/kg chow; n = 14) for 21 d, causing the elimination of approximately 99% of microglia brain-wide. A subset of mice were sacrificed to confirm microglial elimination and are shown in (B) (n = 4). C) For the remaining microglia eliminated mice (n = 10), the inhibitor was removed, allowing new microglia to repopulate the entire CNS during a 21d recovery period. Half brain stitches are shown and each microglia is represented with a white dot. D–F) Images of the hippocampal region for each treatment are shown, with IBA1 staining in green. G–I) 10x and 63x z-stack images of the CA1 hippocampal region for each treatment are shown, with NeuN staining in green and GFAP staining in red.

As we previously demonstrated [30], approximately 99% of microglia were eliminated brain-wide from naïve mice following 21 days of treatment with the CSF1R/c-Kit inhibitor PLX3397 (Fig 1B–1E). However, following removal of the compound, the microglia quickly repopulated the entire brain (Fig 1C–1F), returning to the same number of cells observed in controls (Fig 1A–1D). These data reveal a remarkable capacity for the adult brain to repopulate itself with new cells following nearly complete microglial elimination. The only other known study to eliminate and repopulate microglia used intracerebroventricular ganciclovir treatment in CD11b-HSVTK mice, which express a herpes simplex virus thymidine kinase (HSVTK) under the monocytic CD11b promoter [54]. However, under these conditions, the authors find that repopulation occurred via monocytes from the periphery. In contrast, repopulation following PLX3397 withdrawal in our previous study did not occur from peripheral sources, but instead from a nestin expressing proliferating progenitor cell within the brain itself [30]. The approach used here requires no genetic manipulations or invasive surgeries, but simply the administration of a CSF1R/c-Kit inhibitor in the diet. We previously showed that elimination of microglia had no overt effects on remaining cell types, such as astrocyte numbers, or on neuronal or oligodendrocyte markers [30]. Confocal imaging of astrocytes and neurons in control, microglia depleted, and repopulated tissues confirmed these prior findings (Fig 1G–1I). Despite the loss of over 90% of microglia, inhibitor treatment resulted in no change in stereological volume measurements in the previous study [30].

### Cognitive Behavioral Testing

**Fig 2:**
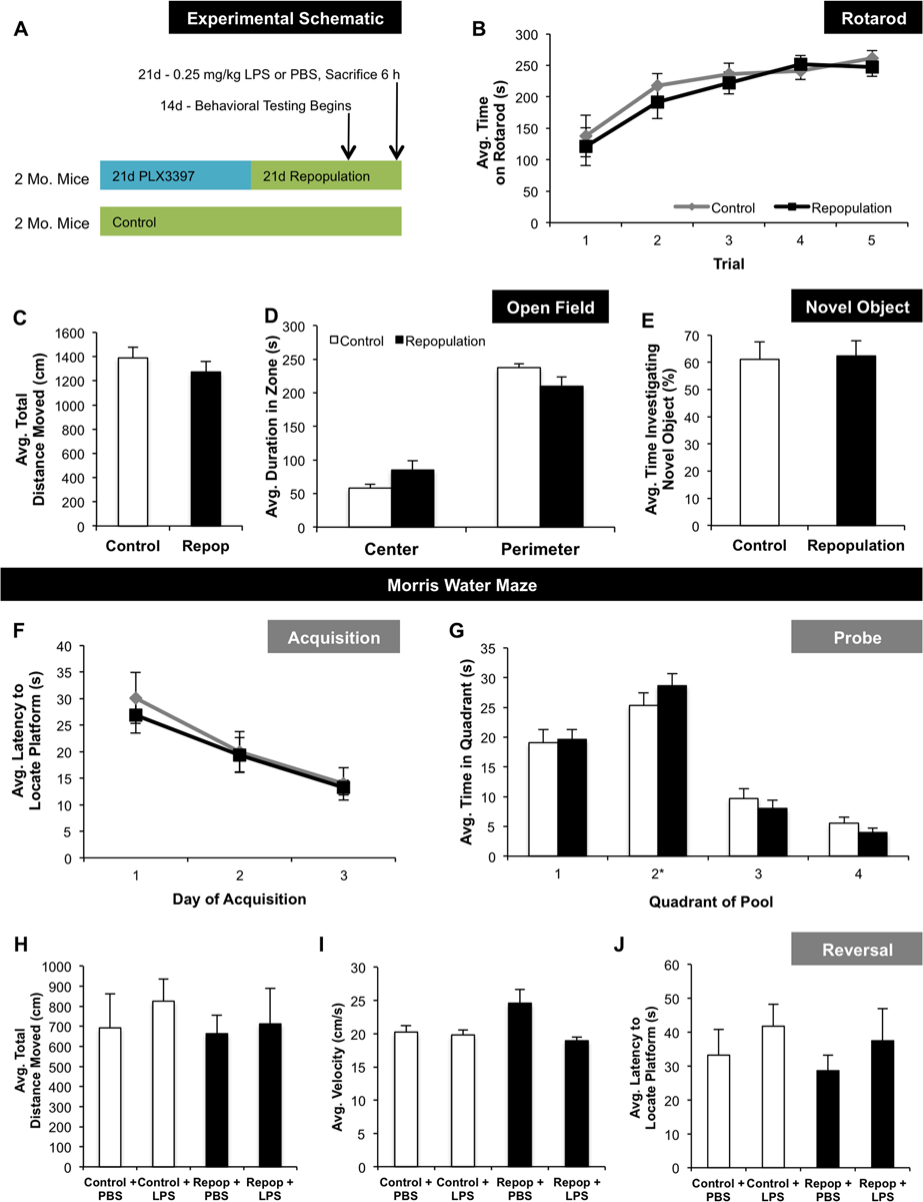
Impacts of Microglial Repopulation on Behavior, Cognition, and Motor Function. A) Schematic describing experimental design. Mice were administered either control diet (n = 10) or PLX3397 for 21 d to eliminate microglia, then the inhibitor was removed to allow microglial repopulation (n = 10). From days 14 to 21, behavioral testing was conducted. Mice were given an LPS challenge (0.25 mg/kg LPS or PBS, both administered IP) on the final day of behavioral testing and sacrificed 6 h later (n = 5 per group). B, C) No changes in motor function were observed, as indicated by time on rotarod and average distance moved in the open field, between control and microglia repopulated animals. D) There were no changes in anxiety between treatments, as assessed by time spent in the center or perimeter of the open field arena. E) Both treatments showed similar cortical learning and memory, as indicated by no difference in the time spent investigating the novel object. F) Control and microglia repopulated mice showed similar learning curves during acquisition of the Morris water maze. G) Both treatments spent similar amounts of time in the different quadrants of the Morris water maze, with the most time spent in the correct quadrant (#2). H–J) Following an LPS challenge, there were no changes in distance moved, velocity, or time to locate the platform during reversal testing (21 d repopulation treatment labeled as “Repop” on figure). Data were analyzed using unpaired student’s t-tests and are presented as raw means ± SEM.

Microglial function can influence cognition and behavior, as well as neuronal structure and function, particularly during diseases and injuries, stimulating chronic neuroinflammation. Elimination of all microglia from the CNS, followed by repopulation, brings about drastic changes to the brain, and could adversely impact behavior and cognition. To address this possibility, mice were treated with PLX3397 for 21 d to eliminate microglia, and then the inhibitor removed to stimulate repopulation. Fourteen days later, a variety of tasks were employed (see experimental schematic; Fig 2A). Microglial repopulation had no effects on motor function, as shown by rotarod testing (Fig 2B) or distance moved in the open field arena (Fig 2C). Repopulation of microglia also had no effect on behavior within the open field arena, where mice spent similar amounts of time in the center and perimeter as control mice (Fig 2D). Microglial repopulation had no effects on learning and memory, as determined by novel object testing (Fig 2E) and Morris water maze (Fig 2F and 2G). During the probe trial, when the platform was removed, but all visual cues remained intact, both controls and mice with newly repopulated microglia spent the most time in the correct quadrant of the pool, demonstrating that they had learned the location of the platform during training (Fig 2G). Finally, LPS was administered to half of the animals from each treatment prior to the start of Morris water maze reversal testing, where the mice were required to learn the new location of the hidden platform, to explore performance in the presence of a neuroinflammatory stimulus. A low dose of LPS (0.25 mg/kg) was chosen, in order to prevent the induction of excessive sickness behavior (e.g., lethargy and reduced interest in the physical environment [55]), which may have prevented the mice from, or reduced their willingness to, perform the reversal task. In addition, the single sub-threshold dose of LPS used in this study was not expected to induce neuronal loss, as evidenced by the literature, whereby a dose of LPS greater than 1 mg/kg or administered repeatedly has been shown to prevent neurogenesis and neuronal survival [11,56,57]. LPS did not impair motor function, as confirmed by total distance moved (Fig 2H) and velocity (Fig 2I), nor the latency to locate the platform in its new location (Fig 2J). These combined data show that newly repopulated microglia do not cause deficits in motor function, behavior, or cognition. Of note, mice lacking microglia during development show impairments in social behavior [58], learning and memory [9,59], and LTP [59]. It may be that the developing brain is particularly sensitive to changes in microglial presence and function, and therefore, eliminating and repopulating microglia at a young age may have more significant impacts on behavior and cognition than in adults. As administration and removal of CSF1R/c-Kit inhibitors to stimulate microglial elimination and repopulation is a potentially translatable therapeutic for the treatment of human disease and disorders, via the potential resolution of inflammation, having no negative impacts on behavior and cognition is an important observation.

### Morphological Analysis of Microglia

**Fig 3:**
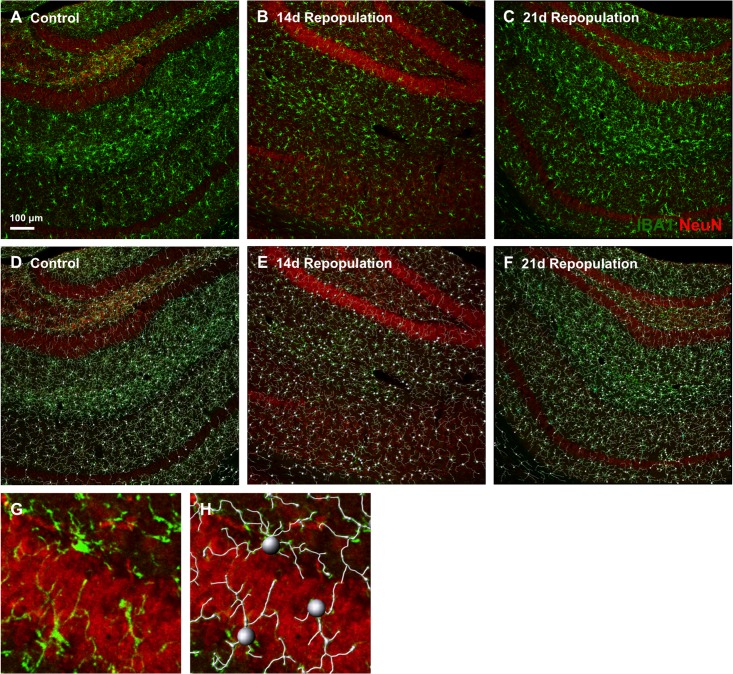
Microglial Modeling for Morphological Analysis. A–G) Examples of microglial modeling in the hippocampus of control, 14 d repopulation, and 21 d repopulation treatments are shown. 10x zoom images were analyzed with Imaris software. NeuN staining is shown in red and IBA1 in green. A–C) Reference images of the hippocampus for each treatment with microglia are shown in green. D–F) Modeling of the microglia with cell bodies and processes traced in white, as determined by the computer software, show nearly identical overlap with the reference images from (A–C). G, H) Zoom image of the microglial modeling from the 14 d repopulation treatment.

**Fig 4:**
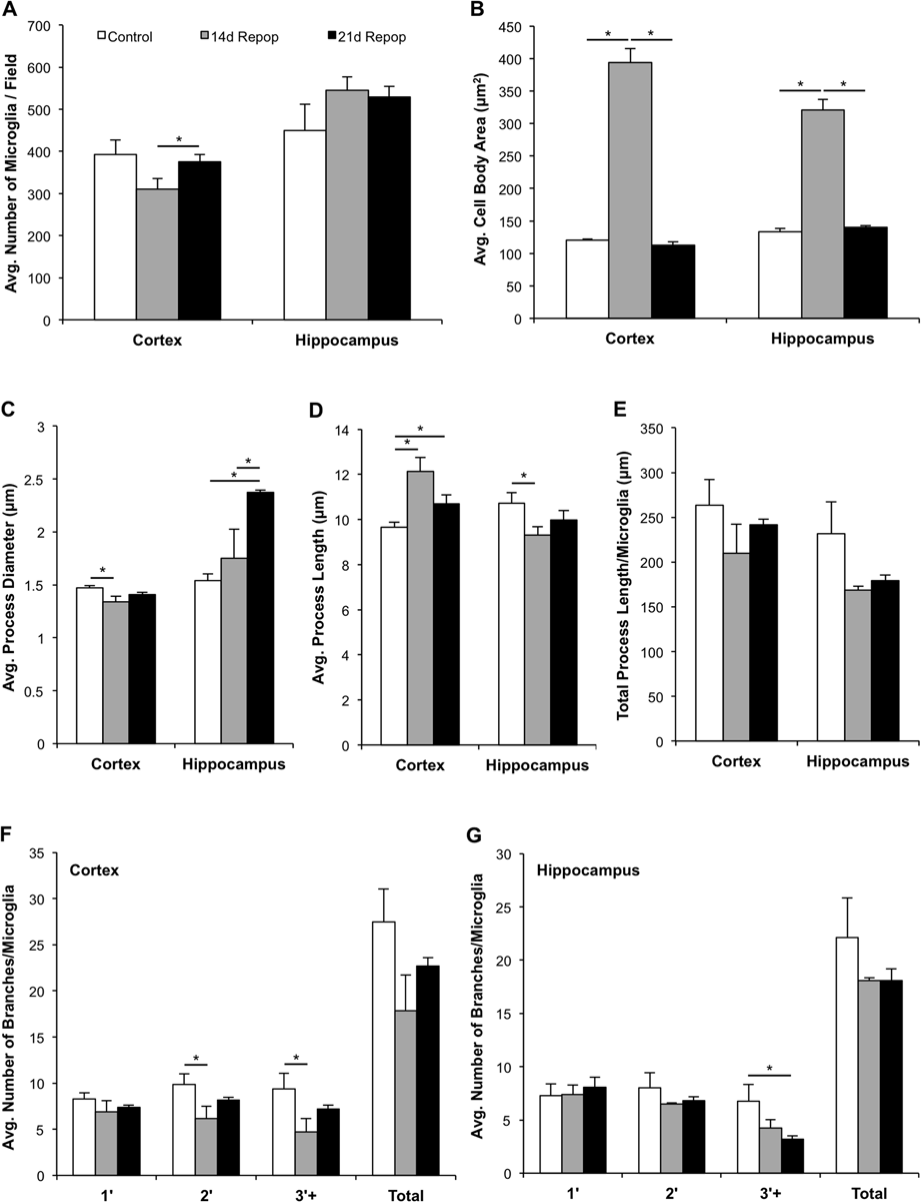
Morphological Analysis of Control vs. Repopulated Microglia. A–G) All morphological markers were examined in control (n = 10), 14 d repopulation (n = 5, “14d Repop”), and 21 d repopulation (n = 10, “21d Repop”) treatments to examine the change in microglia morphology over time. A) Changes in the number of microglia were observed in the cortex, but not the hippocampus during repopulation. B) Cell body area was much larger in 14 d repopulation microglia than either controls or 21 d repopulation in both the cortex and hippocampus. C, D) Microglial process diameter (C) and length (D) varied according to brain region and treatment, with some recovery apparent over time. E) Total process length/microglia did not vary due to brain region or treatment. F, G) The number of primary, secondary, and tertiary (or above) branches/microglia varied due to brain region and treatment, where secondary and tertiary (or above) branches were reduced in the cortex at 14 d repopulation, but had recovered by 21 d repopulation (F). While in the hippocampus, a reduction in the number of tertiary (and above) microglial branches were reduced over the course of repopulation (G). Data were analyzed using unpaired student’s t-tests and are presented as raw means ± SEM. Statistical significance is represented as *P<0.05.

We next set out to characterize the morphology of repopulating microglia and to compare them to resident microglia. Confocal images of brain sections stained with the microglial marker IBA1 were taken and computer software used to model the physical properties of the microglia. We compared resident microglia to repopulated microglia at two different time points (an additional 14 d repopulation group (n = 5) and the original 21 d repopulation group (n = 5)). Examples of the microglial modeling for each treatment are shown in Fig 3, where both the original confocal images of the cells (A-C) and images with the modeling (D-F) are represented. In addition, a close-up of the modeled microglia is shown (Fig 3G and 3H). The number of microglia in the cortex was less than controls at 14 d repopulation, but returned to control levels by 21 d (Fig 4A), while no differences were seen in the hippocampus. Microglia at 14 d repopulation had larger cell bodies (more than twice the size) than controls, which returned to baseline levels by 21 d repopulation (Fig 4B). Notably, within the hippocampus, repopulated microglia had thickened processes compared to control microglia (Fig 4C), but no differences in process length (Fig 4D and 4E). In adult mice, unramified/amoeboid microglia with thickened processes are typically identified as activated/phagocytotic [19,60]. Interestingly, this phenotype is also reminiscent of microglial morphology observed during normal development, and thus microglial repopulation in the adult may recapitulate microglial development in early life [4,5,43,61,62]. Finally, microglial complexity was analyzed by counting the number of primary, secondary, and tertiary (or above) process branches observed/microglia in each brain region. Within the cortex, microglia showed a reduction in secondary and tertiary (and above) branching at 14 d repopulation compared to controls (Fig 4F), but acquired normal complexities by 21 d. In contrast, hippocampal microglia showed reduced tertiary (and above) branching at 21 d repopulation (Fig 4G). A reduced complexity of branching may simply be an early characteristic of these returning cells, similar to adult neurons that differentiate and acquire increased complexity over time post-neurogenesis [63,64]. Overall, it appears that microglia within the cortex and hippocampus do not have identical morphologies during the course of repopulation, highlighting that either the local environment and/or the role of microglia in these brain regions may be different from each other. In the HSVTK model of microglial repopulation, repopulating cells remained morphologically distinct from resident microglia for up to 27 wk after initial repopulation [54]. In contrast, many of the morphological markers assessed in the current study returned to baseline levels within 3 wk of microglial repopulation. The additional time needed for microglia to assume normal morphology in the HSVTK model is likely due to the need for peripheral monocytes to become microglia, while microglial progenitors appear to assume the morphology of resident microglia quite quickly.

### Inflammatory Profiling

**Fig 5:**
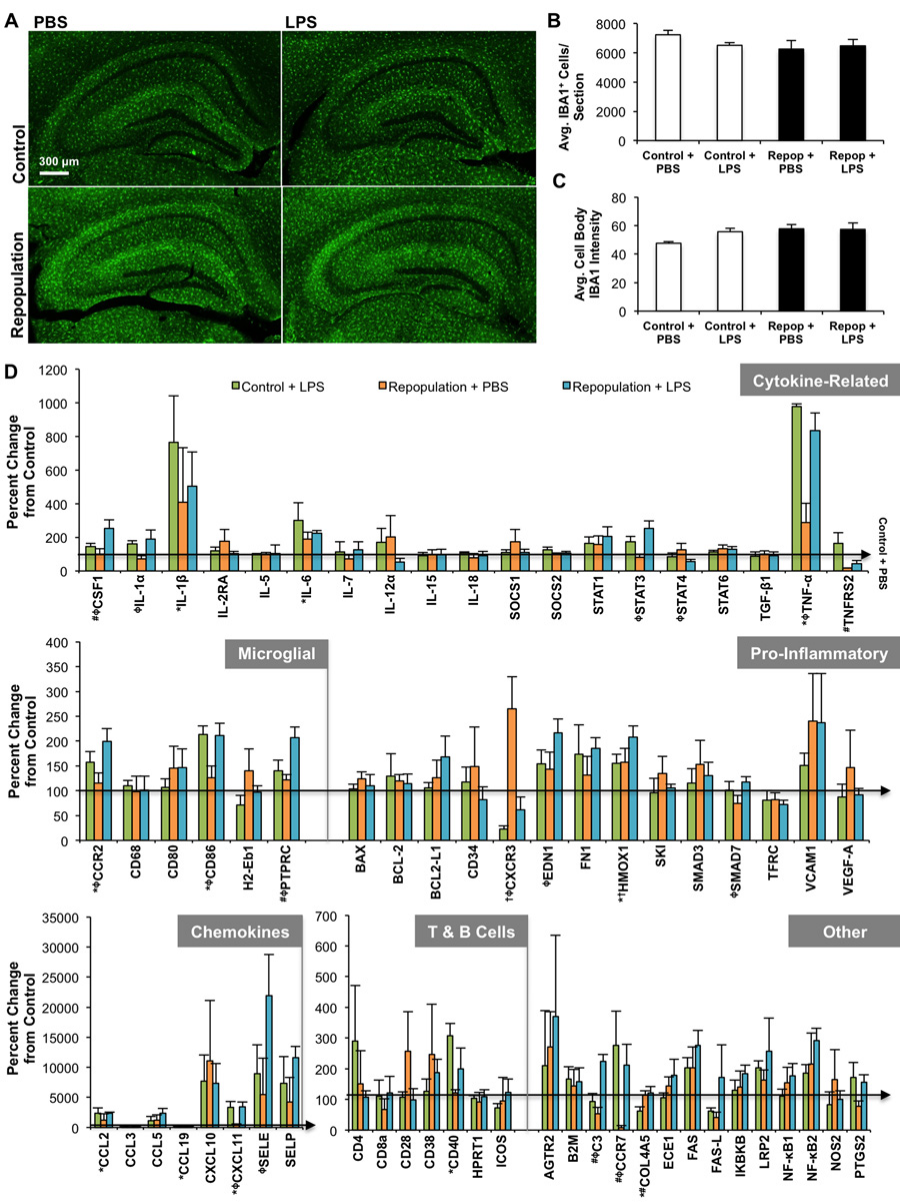
Response of Newly Repopulated Microglia to an Immune Challenge. Half of the mice from each dietary treatment (control or 21 d repopulation, labeled as “Repop” on figure) were administered either LPS (0.25 mg/kg) or an equivalent volume of PBS via IP injection (n = 5 per group). Mice were euthanized 6 h post-injection (following reversal testing in the Morris water maze), half of each brain was collected and snap-frozen for RNA extraction, cDNA was synthesized, and quantitative RT-PCR performed to determine mRNA gene expression for a variety of inflammatory-related markers. A) Images of the hippocampal region of each treatment are shown. B, C) No changes in microglial number or IBA1^+^ staining was observed in response to microglial repopulation or LPS challenge. D) The relative mRNA expression of the microglia repopulated brain in response to LPS for a variety of inflammatory gene markers is shown. Data were analyzed as a two-way ANOVA (control vs. repopulated and LPS vs. PBS) and data are presented as raw means ± SEM. Significance is dictated by the following symbols: control + PBS vs. control + LPS*; control + PBS vs. repopulated + PBS^†^; control + LPS vs. repopulated + LPS^#^; repopulated + PBS vs. repopulated + LPS^ϕ^ (all comparisons, P<0.05). Full P-values can be found in Table 1.

To investigate the ability of the repopulated microglia to respond to an inflammatory challenge, a low dose of LPS was administered. Low doses of LPS have been shown to elicit an inflammatory response in the periphery, as well as the CNS [38,40,65], even in the absence of behavioral/cognitive changes [39,66]. In this study, LPS administration had no impact on microglial number in either the control or repopulated treatments, which were both similar to the PBS controls (Fig 5A–5C). Overall, the mRNA gene expression profile revealed that under normal/resting conditions (PBS administration), repopulated microglia were very similar to resident microglia (Fig 5D). The chemokine receptors *CXCR3* and *HMOX1* were upregulated in response to microglial repopulation; however, all other genes were unchanged. In contrast, when LPS was administered, the repopulated microglia showed a higher number of gene expression changes, including upregulation of *C3*, *COL4A5*, *CSF1*, and *PTPRC* (also known as the *CD45* antigen), but downregulation of *CCR7* and *TNFRS2*. Many of these gene expression changes are associated with an increase in the pro-inflammatory properties of microglia, confirming that these newly repopulated cells are as responsive as resident microglia to an inflammatory agent, and possibly slightly more so. P-values for the data shown in Fig 5D are provided in Table 1.

**Table 1:** P-values for Statistical Analysis of Inflammatory Profiling.

Gene	Diet	Injection	Interaction	CON-PBS vs. CON-LPS (*)	CON-PBS vs. REPOP-PBS (†)	CON-LPS vs. REPOP-LPS (#)	REPOP-PBS vs. REPOP-LPS (ϕ)
**AGTR2**	0.2837	0.4909	0.9672	0.6044	0.4265	0.4597	0.6449
**β2m**	0.6411	0.2924	0.4728	0.1727	0.4468	0.8381	0.8183
**BAX**	0.2337	0.6731	0.4885	0.8451	0.1891	0.7096	0.4331
**BCL-2**	0.9261	0.6088	0.4784	0.3734	0.5836	0.6473	0.8903
**BCL2-L1**	*0*.*0973*	0.3401	0.4699	0.8642	0.4713	*0*.*0972*	0.2422
^**#ϕ**^ **C3**	*0*.*0512*	**0.0018**	**0.0010**	0.7780	*0*.*0988*	**0.0010**	**0.0001**
*CCL2	0.3910	**0.0205**	0.3736	**0.0217**	0.2406	0.9802	0.2513
**CCL3**	0.3018	0.2559	0.5278	0.1724	0.7848	0.1983	0.7296
**CCL5**	0.2801	0.3262	0.6603	0.6566	0.6660	0.2360	0.3603
*CCL19	0.9517	**0.0023**	0.2980	**0.0035**	0.4972	0.4137	*0*.*0792*
* ^**ϕ**^ **CCR2**	0.1277	**0.0021**	0.4652	**0.0363**	0.5562	0.1045	**0.0079**
^**#ϕ**^ **CCR7**	0.1716	**0.0007**	*0*.*0748*	*0*.*0752*	0.7169	**0.0393**	**0.0009**
**CD4**	0.4758	0.4337	0.2202	0.1642	0.6970	0.1781	0.7351
**CD8a**	0.7419	0.3697	0.5536	0.8235	0.5160	0.8505	0.2971
**CD28**	0.2316	0.2252	0.1828	0.9254	*0*.*0830*	0.9151	*0*.*0812*
**CD34**	0.8650	0.5368	0.2994	0.7564	0.3911	0.5316	0.2471
**CD38**	0.1973	0.8326	0.5798	0.8071	0.1965	0.5839	0.5890
*CD40	0.3373	**0.0085**	0.1641	**0.0051**	0.7459	*0*.*0883*	0.2517
**CD68**	0.7743	0.7254	0.8370	0.6824	0.9558	0.7175	0.9206
**CD80**	0.1269	0.8867	0.9059	0.8538	0.2369	0.3038	0.9863
* ^**ϕ**^ **CD86**	0.4868	**0.0001**	0.3956	**0.0004**	0.2979	0.9058	**0.0056**
* ^**#**^ **COL4A5**	**0.0101**	0.2163	*0*.*0862*	**0.0444**	0.4197	**0.0046**	0.6972
^**#ϕ**^ **CSF1**	**0.0026**	**0.0398**	0.3239	0.3843	*0*.*0743*	**0.0052**	**0.0362**
**CXCL10**	0.2780	0.6868	0.2469	0.2536	0.1355	0.9531	0.5926
* ^**ϕ**^ **CXCL11**	0.7711	**0.0012**	0.8539	**0.0054**	0.7517	0.9353	**0.0160**
^**†ϕ**^ **CXCR3**	**0.0058**	**0.0009**	*0*.*0508*	*0*.*0583*	**0.0032**	0.3257	**0.0013**
**ECE1**	**0.0489**	0.4568	0.5984	0.8747	0.2660	*0*.*0774*	0.3726
^**ϕ**^ **EDN1**	**0.0390**	**0.0156**	0.6865	0.1151	0.2031	*0*.*0774*	**0.0414**
**FAS**	**0.0456**	**0.0451**	0.7121	*0*.*0893*	*0*.*0899*	0.2147	0.2135
**FAS-L**	0.6229	0.3612	0.1185	0.5899	0.3965	0.1604	*0*.*0989*
**FN1**	0.5075	*0*.*0692*	0.7717	0.1309	0.5016	0.7894	0.2528
**H2-Eb1**	0.1840	0.1548	0.7723	0.3823	0.2354	0.4548	0.2368
* ^**†**^ **HMOX1**	**0.0087**	**0.0108**	0.9206	**0.0481**	**0.0412**	*0*.*0534*	*0*.*0621*
**HPRT1**	0.9311	0.5179	0.6728	0.8663	0.7286	0.8040	0.4685
**IKBKB**	0.1360	0.2375	0.8368	0.4793	0.3459	0.2251	0.3243
**ICOS**	0.5920	0.9909	0.5152	0.6377	0.9309	0.4204	0.6483
^**ϕ**^ **IL-1α**	0.9799	**0.0088**	0.3142	0.1468	0.4979	0.4434	**0.0149**
*IL-1β	0.9111	0.1061	0.2147	**0.0394**	0.3328	0.4122	0.7714
**IL-2RA**	0.3802	0.4255	0.1919	0.7000	0.1307	0.7443	0.1444
**IL-5**	0.9095	0.9808	0.9080	0.9431	0.9990	0.8673	0.9275
*IL-6	0.8832	**0.0414**	0.1337	**0.0147**	0.2532	0.3027	0.6479
**IL-7**	0.8336	0.3579	0.5517	0.8121	0.5695	0.7834	0.2891
**IL-12α**	0.9098	0.5619	0.1195	0.4598	0.2914	0.2291	0.1340
**IL-15**	0.9008	0.8177	0.8436	0.7532	0.9605	0.8129	0.9818
**IL-18**	0.2579	0.5535	0.8511	0.7635	0.3655	0.4768	0.5950
**LRP2**	0.3432	0.1209	0.9440	0.2399	0.4679	0.5287	0.2778
**NF-κB1**	*0*.*0639*	0.5782	0.8421	0.7991	0.2184	0.1386	0.5937
**NF-κB2**	**0.0422**	0.1175	0.9308	0.2332	0.1207	0.1483	0.2806
**NOS2**	0.3665	0.3732	0.5969	0.7676	0.3304	0.7745	0.3586
**PTGS2**	0.4908	**0.0204**	0.9169	*0*.*0792*	0.5605	0.6855	*0*.*0768*
^**#ϕ**^ **PTPRC**	**0.0141**	**0.0016**	0.1660	*0*.*0764*	0.3504	**0.0079**	**0.0028**
^**ϕ**^ **SELE**	*0*.*0631*	**0.0150**	0.4113	0.1857	0.4139	*0*.*0628*	**0.0229**
**SELP**	0.1599	**0.0222**	0.9824	*0*.*0784*	0.3334	0.2841	*0*.*0949*
**SKI**	0.2800	0.4805	0.5196	0.8936	0.2284	0.7463	0.3036
**SMAD3**	0.2283	0.9004	0.4894	0.6863	0.1863	0.7009	0.5631
^**ϕ**^ **SMAD7**	0.7055	*0*.*0756*	*0*.*0910*	0.9373	0.1552	0.3043	**0.0248**
**SOCS1**	0.2750	0.4040	0.2775	0.8504	0.1327	0.9965	0.1822
**SOCS2**	0.4138	0.1909	0.2773	*0*.*0887*	0.8450	0.1705	0.8674
**STAT1**	0.2086	0.1529	0.8293	0.2405	0.2948	0.4483	0.3740
^**ϕ**^ **STAT3**	0.2679	**0.0009**	0.1003	*0*.*0719*	0.6773	*0*.*0519*	**0.0013**
^**ϕ**^ **STAT4**	0.9433	*0*.*0554*	0.2007	0.5971	0.3828	0.3330	**0.0302**
**STAT6**	*0*.*0840*	0.6646	0.5298	0.4551	*0*.*0987*	0.3989	0.8885
**TFRC**	0.2487	0.2089	0.6830	0.2405	0.2713	0.5849	0.5323
**TGF-β1**	0.9363	0.5373	0.8987	0.5984	0.9733	0.8835	0.7272
* ^**ϕ**^ **TNF-α**	0.7675	**<0.0001**	**0.0472**	**<0.0001**	*0*.*0892*	0.2056	**0.0006**
^**#**^ **TNFRS2**	**0.0180**	0.2481	0.6161	0.1980	0.1670	**0.0246**	0.6569
**VCAM1**	*0*.*0861*	0.6979	0.6580	0.5583	0.1263	0.3366	0.9689
**VEGF-A**	0.4651	0.3493	0.5582	0.7972	0.3563	0.9161	0.2869

Half of the mice from each dietary treatment (control or 21 d repopulated) were administered either LPS (0.25 mg/kg) or an equivalent volume of PBS via IP injection (n = 5 per group) and sacrificed 6 h later. mRNA gene expression for a variety of inflammatory-related markers was investigated. The data were analyzed using a two-way ANOVA (control vs. repopulated and LPS vs. PBS). In the table, the P-values for the main factors (diet and injection), the interaction of these two factors (diet x injection), and the specific post-hoc tests examined (treatment comparisons of interest, Fig 5D) are shown here. Bold font represents statistically significant differences (P<0.05) and italicized font represents statistical trends (P<0.10).

## Conclusions

Overall, our data showed that the newly repopulated microglia caused no cognitive or behavioral impairments, while changes in morphology, including increased cell body size and less complex microglial process branching, appeared to resolve with time. In addition, repopulated microglia appeared functional and responsive to an inflammatory challenge similar to resident microglia. As administration and removal of CSF1R/c-Kit inhibitors to stimulate microglial elimination and repopulation is a potentially translatable therapeutic, having no negative or long lasting impacts on behavior, cell morphology, and neuroinflammation is an important observation. Thus, we have presented data showing that we can eliminate nearly all microglia from adult mice, and then repopulate the entire CNS with newly derived cells that become morphologically and functionally indistinct from resident microglia, opening up many possibilities for treatment and resolution of neuroinflammatory processes.

## References

[1] Kettenmann H, Hanisch U-K, Noda M, Verkhratsky A. Physiology of Microglia. Physiol Rev. 2011;91: 461–553. 10.1152/physrev.00011.2010 21527731

[2] Hanisch U-K, Kettenmann H. Microglia: active sensor and versatile effector cells in the normal and pathologic brain. Nat Neurosci. 2007;10: 1387–1394. 17965659 10.1038/nn1997

[3] Saijo K, Glass CK. Microglial cell origin and phenotypes in health and disease. Nat Rev Immunol. 2011;11: 775–787. 10.1038/nri3086 22025055

[4] Prinz M, Priller J. Microglia and brain macrophages in the molecular age: from origin to neuropsychiatric disease. Nat Rev Neurosci. 2014;15: 300–312. 10.1038/nrn3722 24713688

[5] Harry GJ. Microglia during development and aging. Pharmacol Therapeut. 2013;139: 313–326. 10.1016/j.pharmthera.2013.04.013 PMC3737416 23644076

[6] Ueno M, Yamashita T. Bidirectional tuning of microglia in the developing brain: from neurogenesis to neural circuit formation. Curr Opin Neurobiol. 2014;27C: 8–15. 10.1016/j.conb.2014.02.004 24607651

[7] Paolicelli RC, Bolasco G, Pagani F, Maggi L, Scianni M, Panzanelli P, et al Synaptic pruning by microglia is necessary for normal brain development. Science. 2011;333: 1456–1458. 10.1126/science.1202529 21778362

[8] Schwarz JM, Bilbo SD. Sex, glia, and development: Interactions in health and disease. Horm Behav. 2012;62: 243–253. 10.1016/j.yhbeh.2012.02.018 22387107 PMC3374064

[9] Parkhurst CN, Yang G, Ninan I, Savas JN, Yates JR, Lafaille JJ, et al Microglia promote learning-dependent synapse formation through brain-derived neurotrophic factor. Cell. 2013;155: 1596–1609. 10.1016/j.cell.2013.11.030 24360280 PMC4033691

[10] Kettenmann H, Kirchhoff F, Verkhratsky A. Microglia: new roles for the synaptic stripper. Neuron. 2013;77: 10–18. 10.1016/j.neuron.2012.12.023 23312512

[11] Kohman RA, Rhodes JS. Neurogenesis, inflammation and behavior. Brain, behavior, and immunity. 2013;27: 22–32. 10.1016/j.bbi.2012.09.003 22985767 PMC3518576

[12] Blank T, Prinz M. Microglia as modulators of cognition and neuropsychiatric disorders. Glia. 2013;61: 62–70. 10.1002/glia.22372 22740320

[13] Neumann H, Wekerle H. Brain microglia: Watchdogs with pedigree. Nat Neurosci. 2013;16: 253–255. 10.1038/nn.3338 23434975

[14] Ginhoux F, Greter M, Leboeuf M, Nandi S, See P, Gokhan S, et al Fate mapping analysis reveals that adult microglia derive from primitive macrophages. Science. 2010;330: 841–845. 10.1126/science.1194637 20966214 PMC3719181

[15] Jenkins SJ, Hume DA. Homeostasis in the mononuclear phagocyte system. Trends Immunol. 2014;35: 358–367. 10.1016/j.it.2014.06.006 25047416

[16] Ramlackhansingh AF, Brooks DJ, Greenwood RJ, Bose SK, Turkheimer FE, Kinnunen KM, et al Inflammation after trauma: microglial activation and traumatic brain injury. Ann Neurol. 2011;70: 374–383. 10.1002/ana.22455 21710619

[17] Koshinaga M, Katayama Y, Fukushima M, Oshima H, Suma T, Takahata T. Rapid and widespread microglial activation induced by traumatic brain injury in rat brain slices. J Neurotraum. 2000;17: 185–192. 10.1089/neu.2000.17.185 10757324

[18] Dilger RN, Johnson RW. Aging, microglial cell priming, and the discordant central inflammatory response to signals from the peripheral immune system. J Leukocyte Biol. 2008;84: 932–939. 10.1189/jlb.0208108 18495785 PMC2538600

[19] Neumann H, Kotter MR, Franklin RJM. Debris clearance by microglia: an essential link between degeneration and regeneration. Brain. 2009;132: 288–295. 10.1093/brain/awn109 18567623 PMC2640215

[20] Mosher KI, Wyss-Coray T. Microglial dysfunction in brain aging and Alzheimer's disease. Biochem Pharmacol. 2014;88: 594–604. 10.1016/j.bcp.2014.01.008 24445162 PMC3972294

[21] Cunningham C. Microglia and neurodegeneration: the role of systemic inflammation. Glia. 2013;61: 71–90. 10.1002/glia.22350 22674585

[22] Doens D, Fernández PL. Microglia receptors and their implications in the response to amyloid β for Alzheimer's disease pathogenesis. J Neuroinflamm. 2014;11: 48. 10.1186/1742-2094-11-48 PMC3975152 24625061

[23] Serpente M, Bonsi R, Scarpini E, Galimberti D. Innate immune system and inflammation in Alzheimer's disease: from pathogenesis to treatment. Neuroimmunomodulat. 2014;21: 79–87. 10.1159/000356529 24557039

[24] Hume D, MacDonald KPA. Therapeutic applications of macrophage colony-stimulating factor-1 (CSF-1) and antagonists of CSF-1 receptor (CSF-1R) signaling. Blood. 2012;119: 1810–1820. 10.1182/blood-2011-09-379214 22186992

[25] Patel S, Player MR. Colony-stimulating factor-1 receptor inhibitors for the treatment of cancer and inflammatory disease. Curr Top Med Chem. 2009;9: 599–610. 19689368 10.2174/156802609789007327

[26] Nandi S, Gokhan S, Dai X-M, Wei S, Enikolopov G, Lin H, et al The CSF-1 receptor ligands IL-34 and CSF-1 exhibit distinct developmental brain expression patterns and regulate neural progenitor cell maintenance and maturation. Dev Biol. 2013;367: 100–113. 10.1016/j.ydbio.2012.03.026 PMC3388946 22542597

[27] Erblich B, Zhu L, Etgen AM, Dobrenis K, Pollard JW. Absence of colony stimulation factor-1 receptor results in loss of microglia, disrupted brain development and olfactory deficits. Plos One. 2011;6: e26317 10.1371/journal.pone.0026317 22046273 PMC3203114

[28] Rademakers R, Baker M, Nicholson AM, Rutherford NJ, Finch N, Soto-Ortolaza A, et al Mutations in the colony stimulating factor 1 receptor (CSF1R) gene cause hereditary diffuse leukoencephalopathy with spheroids. Nat Genet. 2012;44: 200–205. 10.1038/ng.1027 22197934 PMC3267847

[29] Nicholson AM, Baker MC, Finch NA, Rutherford NJ, Wider C, Graff-Radford NR, et al CSF1R mutations link POLD and HDLS as a single disease entity. Neurology. 2013;80: 1033–1040. 10.1212/WNL.0b013e31828726a7 23408870 PMC3653204

[30] Elmore MRP, Najafi AR, Koike MA, Dagher NN, Spangenberg EE, Rice RA, et al Colony-stimulating factor 1 receptor signaling is necessary for microglia viability, unmasking a cell that rapidly repopulates the microglia-depleted adult brain. Neuron. 2014;82: 380–397. 10.1016/j.neuron.2014.02.040 24742461 PMC4161285

[31] Abou-Khalil R, Yang F, Mortreux M, Lieu S, Yu Y-Y, Wurmser M, et al Delayed bone regeneration is linked to chronic inflammation in murine muscular dystrophy. J Bone Miner Res. 2014;29: 304–315. 10.1002/jbmr.2038 23857747 PMC3893315

[32] Chitu V, Nacu V, Charles JF, Henne WM, Mcmahon HT, Nandi S, et al PSTPIP2 deficiency in mice causes osteopenia and increased differentiation of multipotent myeloid precursors into osteoclasts. Blood. 2012;120: 3126–3135. 10.1182/blood-2012-04-425595 22923495 PMC3471520

[33] Coniglio SJ, Eugenin E, Dobrenis K, Stanley ER, West BL, Symons MH, et al Microglial stimulation of glioblastoma invasion involves epidermal growth factor receptor (EGFR) and colony stimulating factor 1 receptor (CSF-1R) signaling. Mol Med. 2012;18: 519–527. 10.2119/molmed.2011.00217 22294205 PMC3356419

[34] DeNardo DG, Brennan DJ, Rexhepaj E, Ruffell B, Shiao SL, Madden SF, et al Leukocyte complexity predicts breast cancer survival and functionally regulates response to chemotherapy. Cancer Discov. 2011;1: 54–67. 10.1158/2159-8274.CD-10-0028 22039576 PMC3203524

[35] He Y, Rhodes SD, Chen S, Wu X, Yuan J, Yang X, et al c-Fms signaling mediates neurofibromatosis Type-1 osteoclast gain-in-functions. Plos One. 2012;7: e46900 10.1371/journal.pone.0046900 23144792 PMC3492362

[36] Mok S, Koya RC, Tsui C, Xu J, Robert L, Wu L, et al Inhibition of CSF-1 receptor improves the antitumor efficacy of adoptive cell transfer immunotherapy. Cancer Res. 2014;74: 153–161. 10.1158/0008-5472.CAN-13-1816 24247719 PMC3947337

[37] Prada CE, Jousma E, Rizvi TA, Wu J, Dunn RS, Mayes DA, et al Neurofibroma-associated macrophages play roles in tumor growth and response to pharmacological inhibition. Acta Neuropathol. 2013;125: 159–168. 10.1007/s00401-012-1056-7 23099891 PMC3547628

[38] Godbout JP, Chen J, Abraham J, Richwine AF, Berg BM, Kelley KW, et al Exaggerated neuroinflammation and sickness behavior in aged mice after activation of the peripheral innate immune system. FASEB J. 2005;24: 1–24. 10.1096/fj.05-3776fje 15919760

[39] Chen J, Buchanan JB, Sparkman NL, Godbout JP, Freund GG, Johnson RW. Neuroinflammation and disruption in working memory in aged mice after acute stimulation of the peripheral innate immune system. Brain, behavior, and immunity. 2008;22: 301–311. 17951027 10.1016/j.bbi.2007.08.014 PMC2374919

[40] Henry CJ, Huang Y, Wynne AM, Godbout JP. Peripheral lipopolysaccharide (LPS) challenge promotes microglial hyperactivity in aged mice that is associated with exaggerated induction of both pro-inflammatory IL-1 beta and anti-inflammatory IL-10 cytokines. Brain Behavior and Immunity. 2009;23: 309–317. 10.1016/j.bbi.2008.09.002 18814846 PMC2692986

[41] Püntener U, Booth SG, Perry VH, Teeling JL. Long-term impact of systemic bacterial infection on the cerebral vasculature and microglia. J Neuroinflamm. 2012;9: 146. 10.1186/1742-2094-9-146 PMC3439352 22738332

[42] Yirmiya R, Goshen I. Immune modulation of learning, memory, neural plasticity and neurogenesis. Brain, behavior, and immunity. 2011;25: 181–213. 10.1016/j.bbi.2010.10.015 20970492

[43] Bilbo SD, Schwartz JM. Early-life programming of later-life brain and behavior: A critical role for the immune system. Front Behav Neurosci. 2009;3: 1–14. 10.3389/neuro.08.001.2009 19738918 PMC2737431

[44] Simon P, Dupuis R, Costentin J. Thigmotaxis as an index of anxiety in mice. Influence of dopaminergic transmissions. Behav Brain Res. 1994;61: 59–64. 7913324 10.1016/0166-4328(94)90008-6

[45] Lamprea MR, Cardenas FP, Setem J, Morato S. Thigmotactic responses in an open-field. Braz J Med Biol Res. 2008;41: 135–140. 18297193 10.1590/s0100-879x2008000200010

[46] Treit D, Fundytus M. Thigmotaxis as a test for anxiolytic activity in rats. Pharmacol Biochem Be. 1988;31: 959–962. 3252289 10.1016/0091-3057(88)90413-3

[47] Vorhees CV, Williams MT. Morris water maze: Procedures for assessing spatial and related forms of learning and memory. Nat Protoc. 2006;1: 848–858. 17406317 10.1038/nprot.2006.116 PMC2895266

[48] D'Hooge R, De Deyn PP. Applications of the Morris water maze in the study of learning and memory. Brain Res Rev. 2001;36: 60–90. 11516773 10.1016/s0165-0173(01)00067-4

[49] Morris R. Developments of a water-maze procedure for studying spatial learning in the rat. J Neurosci Meth. 1984;11: 47–60. 10.1016/0165-0270(84)90007-4 6471907

[50] Jurgens HA, Amancherla K, Johnson RW. Influenza infection induces neuroinflammation, alters hippocampal neuron morphology, and impairs cognition in adult mice. The Journal of Neuroscience. 2012;32: 3958–3968. 10.1523/JNEUROSCI.6389-11.2012 22442063 PMC3353809

[51] Neely KM, Green N, Laferla FM. Presenilin is necessary for efficient proteolysis through the autophagy-lysosome in a gamma-secretase independent manner. The Journal of Neuroscience. 2011;31: 2781–2791. 10.1523/JNEUROSCI.5156-10.2010 21414900 PMC3064964

[52] Livak KJ, Schmittgen TD. Analysis of relative gene expression data using real-time quantitative PCR and the 2(T)(-Delta Delta C) method. Methods. 2001;25: 402–408. 11846609 10.1006/meth.2001.1262

[53] Grafen A, Hails R. Modern Statistics for the Life Sciences. 2002.

[54] Varvel NH, Grathwohl SA, Baumann F, Liebig C, Bosch A, Ransohoff RM, et al Microglial repopulation model reveals a robust homeostatic process for replacing CNS myeloid cells. PNAS. 2012: 2–7. 10.1073/pnas.1210150109 PMC3497743 23071306

[55] Dantzer R, O'Connor JC, Freund GG, Johnson RW, Kelley KW. From inflammation to sickness and depression: When the immune system subjugates the brain. Nat Rev Neurosci. 2008;9: 46–57. 18073775 10.1038/nrn2297 PMC2919277

[56] Monje ML, Toda H, Palmer TD. Inflammatory blockade restores adult hippocampal neurogenesis. Science. 2003;302: 1760–1765. 14615545 10.1126/science.1088417

[57] Bastos GN, Moriya T, Inui F, Katura T, Nakahata N. Involvement of cyclooxygenase-2 in lipopolysaccharide-induced impairment of the newborn cell survival in the adult mouse dentate gyrus. Neuroscience. 2008;155: 454–462. 10.1016/j.neuroscience.2008.06.020 18616986

[58] Zhan Y, Paolicelli RC, Sforazzini F, Weinhard L, Bolasco G, Pagani F, et al Deficient neuron-microglia signaling results in impaired functional brain connectivity and social behavior. Nat Neurosci. 2014;17: 400–406. 10.1038/nn.3641 24487234

[59] Rogers JT, Morganti JM, Bachstetter AD, Hudson CE, Peters MM, Grimmig BA, et al CX3CR1 deficiency leads to impairment of hippocampal cognitive function and synaptic plasticity. The Journal of Neuroscience. 2011;31: 16241–16250. 10.1523/JNEUROSCI.3667-11.2011 22072675 PMC3236509

[60] Lynch MA. The multifaceted profile of activated microglia. Mol Neurobiol. 2009;40: 139–156. 10.1007/s12035-009-8077-9 19629762

[61] Perry VH, Hume DA, Gordon S. Immunohistochemical localization of macrophages and microglia in the adult and developing mouse brain. Neuroscience. 1985;15: 313–326. 3895031 10.1016/0306-4522(85)90215-5

[62] Ling EA, Wong WC. The origin and nature of ramified and amoeboid microglia: a historical review and current concepts. Glia. 1993;7: 9–18. 8423067 10.1002/glia.440070105

[63] Espósito MS, Piatti VC, Laplagne DA, Morgenstern NA, Ferrari CC, Pitossi FJ, et al Neuronal differentiation in the adult hippocampus recapitulates embryonic development. The Journal of Neuroscience. 2005;25: 10074–10086. 16267214 10.1523/JNEUROSCI.3114-05.2005 PMC6725804

[64] Mongiat LA, Schinder AF. Adult neurogenesis and the plasticity of the dentate gyrus network. Eur J Neurosci. 2011;33: 1055–1061. 10.1111/j.1460-9568.2011.07603.x 21395848

[65] Burton MD, Johnson RW. Interleukin-6 trans-signaling in the senescent mouse brain is involved in infection-related deficits in contextual fear conditioning. Brain, behavior, and immunity. 2012;26: 732–738. 10.1016/j.bbi.2011.10.008 22062497 PMC3699311

[66] Godbout JP, Moreau M, Lestage J, Chen J, Sparkman NL, O'Connor J, et al Aging exacerbates depressive-like behavior in mice in response to activation of the peripheral innate immune system. Neuropsychopharmacology: official publication of the American College of Neuropsychopharmacology. 2008;33: 2341–2351. 18075491 10.1038/sj.npp.1301649 PMC2907915

